# Antioxidant Evaluation of Extracts of Pecan NutShell (*Carya illinoensis*) in Soybean Biodiesel B100

**DOI:** 10.1002/gch2.201900001

**Published:** 2019-09-02

**Authors:** Alexandra Aparecida do Amaral, Geciele Caroline Schuster, Nayara Lais Boschen, Dalila Moter Benvegnú, Jair Wyzykowski, Paulo Rogério Pinto Rodrigues, André Lazarin Gallina

**Affiliations:** ^1^ Federal University of Fronteira Sul Realeza 85770‐000 Paraná Brazil; ^2^ State University of Central‐West ‐ UNICENTRO Guarapuava 85040‐080 Paraná Brazil; ^3^ Federal University of the Recôncavo of Bahia ‐ UFRB Cruz das Almas 44380‐000 Bahia Brazil

**Keywords:** biofuels, innovation, kinetics, oxidation, rancimat

## Abstract

Biodiesel is subject to radical reactions that promote degradation. To decrease the speed of these degradation reactions and increase oxidative stability, either natural or synthetic antioxidants are added to biodiesel. Thus, the objective of this study is to evaluate the effect of extracts of pecan nutshell (*Carya illinoensis*) as natural antioxidants derived from biomass using water, ethanol, and methanol/water (50/50) as a solvent for extraction. The addition of these antioxidants is performed during the soybean biodiesel washing process in an unconventional manner. The results obtained are statistically analyzed and compared to the control. The induction time (IT) for the biodiesel samples washed with ethanolic extract of pecan nutshell (5 g L^−1^), aqueous extract of pecan nutshell (12 g L^−1^) and methanol/water extract of pecan nutshell (12 g L^−1^), are, respectively, 9.46, 7.60, 7.43 h. The activation energy and the storage time of the biodiesel samples washed with the extracts are also studied. The order of reaction of the oxidation kinetics of biodiesel is first order.

## 1 Introduction

In the last decades, a population growth and technological development has been observed, which is characterized by the increase in energy consumption as a way of solving world needs for comfort and mobility.^1^ Currently, most of the energy consumed and produced in the world originates from fossil sources, highlighting the fossil fuels that are nonrenewable energy sources and can be exhausted.^1^, ^2^, ^3^ In addition, the burning of these fuels produces gases and toxic substances that pollute the atmosphere and harm the health of living beings, being mainly responsible for the greenhouse effect, acid rain and global warming.^2^

Therefore, a promising alternative found to alleviate such problems and minimize dependence on fossil fuels is the production of biofuels. Biofuels are fuels derived from renewable biomass, which can partially or totally replace fossil fuels.^4^ In addition to contributing to the environment by not emitting toxic and polluting compounds during combustion, such as sulfur dioxide (SO_2_) and nitrogen dioxide (NO_2_), and by providing cleaner production, biofuels offer social actions and stimulate economic development and sustainable to the countries that adopt them in their energy matrix.^5^

The production of biodiesel, besides being environmentally correct because it has characteristics of not being a toxic fuel, has a low rate of gas emissions and presents a partially closed carbon cycle. In this cycle, most of the carbon dioxide released during the combustion of biodiesel is absorbed in the process of photosynthesis of oil plants that will be used as raw material for the production of biodiesel.^3^ This biofuel contributes to the sustainable development of family agriculture, to generate income in the poorer parts of Brazilian agriculture, allowing the use of unattractive and remote areas. This makes it a viable alternative socially and economically.^6^

**Scheme 1:**
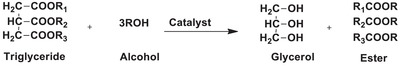
Reaction of transesterification of soybean oil.

Biodiesel can be produced by some methods such as cracking, esterification or transesterification. The latter process is currently the most used for the production of this biofuel because it is more efficient and low cost.^2^ In transesterification there is a reaction between vegetable oils and/or animal fat, with an alcohol, methane or ethanol, in the presence of a catalyst,^3^, ^7^ producing a mixture of methyl esters (biodiesel) and glycerol as a co‐product.^3^ A transesterification reaction is presented in **Scheme** **1**.

The efficiency of the transesterification reaction varies according to some factors, such as the type of alcohol and catalysis (acid and alkaline) used, as well as the size of the carbonic chain of the oil chosen for biodiesel production. Alkaline catalysis (KOH) is more commonly used because promotes a faster reaction and with higher yields.^2^ Regardless of the aforementioned factors, in order for biodiesel to be commercialized, strict quality control is required, which are regulated by the National Agency for Petroleum, Natural Gas, and Biofuels—ANP.^3^, ^7^ Among the quality control tests that are carried out for biodiesel, oxidation stability is the subject of numerous researches, since it is a comparison parameter used to determine the oxidation resistance and guarantee the biodiesel quality.^7^, ^8^

The oxidation of biodiesel occurs, because this biofuel, unlike the fossil fuels that are chemically more stable, is subject to degradation reaction.^3^ Regardless of the raw material used in the production of biodiesel, this tends to the degradation reaction, because the fatty acids nature of the oils and fats used in the production of biodiesel favors the development of oxidation due to its high instauration.^7^, ^8^, ^9^ Moreover, after the production of biodiesel, it is stored until it is consumed and during that period the contact of biodiesel with the metal of the reservoir (carbon steel) or with air oxygen, light, temperature, free radicals, and the inorganic and microbial contaminants trigger radical reactions that promote the degradation of this biofuel, providing the formation of peroxide, hydroperoxides and affecting oxidative stability.^3^, ^5^, ^9^

There are several techniques to measure the oxidation of Biodiesel, in which these accelerated oxidation experiments indicate the induction time (IT), which is the period in which the sample is considered stable in the relation of oxidation.^9^, ^10^ Then, as of November 2014, oxidation stability at 110 °C has its minimum limit of 9 h.^11^

Thus, oxidation stability is an important criterion that must be evaluated in biodiesel qualification, due to the numerous consequences that the degradation of biodiesel provides.^3^, ^7^, ^8^ To minimize the degradation reaction rate and increase the oxidation stability of the biodiesel, antioxidants are added to it.^9^, ^10^, ^12^, ^13^, ^14^

The addition of an antioxidant to biodiesel, which may be natural or synthetic, has the function of reacting with the radicals formed in the initiation and propagation stages, minimizing the number of free radicals or in some cases extinguishing them. These substances are the object of studies that aim to increase the time of induction of biodiesel.^9^, ^10^, ^12^, ^13^, ^14^

The greatest advantage of the use of natural antioxidants is related to their less toxicity in relation to synthetic antioxidants such as *tert*‐butyl hydroquinone (TBHQ). Faced with such a scenario, the natural antioxidants that are derived from biomass and not from oil as the synthetic antioxidants, are gaining the focus of several scientific research. One of these antioxidants is the extract of pecan nutshell [*Carya illinoinensis* (Wangenh) C. Kock].

The pecan nutshell originates from the south of the United States and has the high antioxidant capacity, besides having unsaturated fatty acids. The nut processing for the production of oil and other derivatives produces about 50% of bark, which is considered a by‐product.^15^

The pecan nutshell has been the subject of studies in the alimentary and pharmacological area because it presents antioxidant effects, being recommended as a herbal medicine because it presents health benefits.^16^ Recent studies involving the aqueous extract of pecan nutshell, tested in rats, on the toxicity induced by cyclophosphamide, point to its use as antioxidant against free radicals produced by cancer treatment.^15^ The aqueous extract of the pecan nutshell was also used in hepatoprotective activity to minimize the effects caused by oxidative stress in the liver of rats, caused by the ingestion of ethanol.^17^ The same treatment with the extract aided in oxidative lesions induced by exposure to cigarette smoke, with beneficial effects.^18^

According to Prado et al., the ethanolic extract of pecan nutshell can be used as an antioxidant for linoleic acid, obtaining results with 90% efficiency in the reduction of oxidative processes. In this same work, the extracts of the pecan pie were tested and the aqueous extract from the pecan nutshell and the results with respect to inhibition of the oxidation process were significant.^19^

Based on the literature, there are still no studies that accurately report the antioxidant substance, are the bioactive compounds that are present in the pecan nutshell. However, according to Prado et al., the following major phenolic compounds present in the shell were identified and quantified by high performance liquid chromatography (HPLC): gallic acid, chlorogenic acid, p‐hydroxybenzoic acid, epicatechin gallate, and ellagic acid.^20^

It should be noted that there are no scientific papers and patents that involve the pecan nutshell extract as an antioxidant for soybean biodiesel, so the study of this perspective is quite interesting. In this sense, the objective of this work was to evaluate the antioxidant action of extracts of pecan nutshell on soybean B100, in order to increase their oxidative stability.

## 2 Results and Discussion

### 2.1 Oxidative Stability

The values obtained in the oxidation stability test (IT) for B100 soybean biodiesel washed in a conventional manner (control) and the samples washed with the pecan nutshell extracts are shown in **Table** **1**.

**Table 1:** Induction time for soybean biodiesel washed with pecan nutshell extracts in the conventional manner (control)

Biodiesel washing^a)^	Concentration [g L^−1^]	Induction time–IT [h]^b)^, ^c)^
ANE5	5	3.21 ± 0.11^c)^
ANE10	10	5.08 ± 0.21^b)^, ^c)^
ANE15	15	4.79 ± 0.73^b)^, ^c)^
ENE5	5	9.93 ± 0.84^a)^
ENE10	10	7.69 ± 1.40^a)^, ^b)^
ENE15	15	7.43 ± 1.59^a)^, ^b)^
CONTROL1	0	4.91 ± 1.13^b)^, ^c)^
WNE5	5	5.00 ± 0.50^a)^
WNE10	10	6.14 ± 0.42^a)^
WNE15	15	4.93 ± 0.22^a)^
CONTROL2	0	0.59 ± 0.35^b)^

^a)^ Control 1 was used for extracts of pecan nutshell with water and ethanol solvents. Control 2 was used for the pecan nutshell extracts with methanol + water solvent. Acronyms were established for each extract in which the first letter refers to the type of solvent used (A for aqueous, E for ethanolic, and M for methanol + water), the second letter means pecan nutshell (N) and the third letter means extract (E). The number that accompanies the acronym indicates the concentration of the extract in g L^−1^. It should be noted that control 1 was used for the comparison of ethanolic and aqueous pecan nutshell extracts. The control 2 was used to compare pecan nutshell extract methanol + water

^b)^ Means ± standard deviation

^c)^ Means followed by the same letter in the columns do not differ by Scott‐Knott test (95% significance).

According to the results of Table 1, it can be observed that there is a difference between the types of biodiesel washing, which were used, with the respective controls. This indicates that the presence of the extracts during this washing process modified the IT. However, some IT values were close to the values obtained for the control and for the other concentrations used in the extracts of the pecan nutshell. Thus, it was necessary to obtain a response with more precision so that it can be affirmed if there is a difference between washing the biodiesel in the presence of substances that contain the antioxidants or not.

In this way, the IT data were submitted to a statistical analysis in order to verify the washes of the biodiesel with the extracts that were shown with the best IT results and which presented differences in relation to the control. According to the Scott‐Knott test at 5% significance, the residues from the analysis of variance can be considered normal.

At the end of the statistical analysis, it is possible to select, for each type of solvent used to obtain the extracts, a concentration that provided the highest IT average of the unconventionally washed biodiesel samples, being considered statistically different from the control. The selected concentrations were 5 g L^−1^ for ethanolic pecan nutshell extract (9.93 h) and 10 g L^−1^ for methanol + water pecan nutshell extract (50/50) (6.14 h).

**Figure 1:**
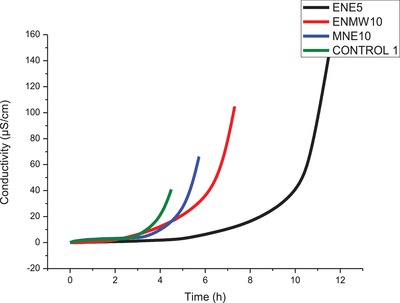
Graphic of Conductivity curves versus time to obtain the induction time for the B100 soybean biodiesel washed in a conventional manner (control 1) and the extracts of the pecan nutshell that had the highest induction time.

Regarding the concentration of aqueous pecan nutshell extract, 10 g L^−1^ (5.08 h) was selected regardless of whether it was not considered statistically different from the control, but because it had a higher average of IT, among the extracts tested. These differences can be seen in the graph of **Figure** **1**. Figure 1 shows the results of the conductivity (µS cm^−1^) in function of time (h) for the biodiesel samples washed with the pecan shell extracts that were statistically different from the control and for the control itself.

It should be noted that IT is obtained by inflecting the conductivity curves and that from Figure 1. It is clearly observed that these times are distinct between the samples. From these preliminary tests, extracts with new concentrations were obtained, according to **Table** **2**, considering the concentration of the pecan nutshell used in the biodiesel washing that provided the highest IT in the previous tests as a central point, varying two concentration units for more and two units for less, in order to optimize the results. It should be noted that IT is obtained by inflecting the conductivity curves and that from Figure 1 it is clearly observed that these times are distinct between the samples. From these preliminary tests, extracts with new concentrations were obtained, according to Table 2, considering the concentration of the pecan nutshell used in the biodiesel washing that provided the highest IT in the previous tests as a central point, varying two concentration units for more and two units for less, in order to optimize the results.

**Table 2:** Induction time for soybean biodiesel washed with pecan nutshell extracts in the conventional manner (control 3)

Biodiesel washing^a)^	Concentration [g L^−1^]	Induction time–IT [h]^b)^, ^c)^
ANE8	8	7.38 ± 0.05^b)^
ANE10	10	5.08 ± 0.21^d,e)^
ANE12	12	7.40 ± 0.27^b)^
ENE3	3	4.68 ± 0.62^e)^
ENE5	5	9.45 ± 0.01^a)^
ENE7	7	4.81 ± 0,26^d,e)^
WNE8	8	7.08 ± 0.40^b)^, ^c)^
WNE10	10	6.14 ± 0.26^c)^
WNE12	12	7.37 ± 0.08^b)^, ^c)^
CONTROL3	0	5.59 ± 0.23^d,e)^

^a)^ Acronyms were established for each extract in which the first letter refers to the type of solvent used (A for aqueous, E for ethanolic, and M for methanol + water), the second letter means pecan nutshell (N), and the third letter means extract (E). The number that accompanies the acronym indicates the concentration of the extract in g L^−1^. It should be noted that control 1 was used for the comparison of ethanolic and aqueous pecan nutshell extracts. The control 2 was used to compare pecan nutshell extract methanol + water

^b)^ Means ± standard deviation

^c)^ Means followed by the same letter in the columns do not differ by Scott‐Knott test (95% significance).

**Figure 2:**
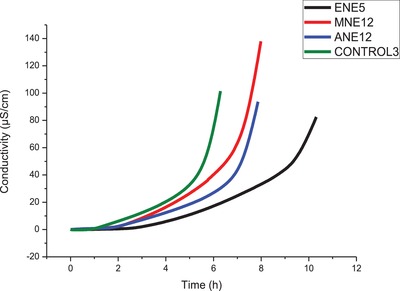
Graphic of conductivity curves versus time to obtain the induction time for the B100 soybean biodiesel washed in a conventional manner (control 3) and the extracts of the pecan nutshell that had the highest induction time.

It is observed in Table 2 that, just as in the previous samples of the optimization, there is a difference between the types of biodiesel washing that were used. In order to have a more precise analysis of which washes types that obtained significant results, a statistical study of comparison of means was also performed. According to Table 2, the biodiesel samples washed with the extracts of the pecan nutshell that presented the highest average of IT and are different from the control were then washed with ANE12 (7.40 h), with ENE5 (9.45 h) and MNE12 (7.37 h). These results are clearly observed in **Figure** **2**, where the graph of the conductivity curves versus the time of these extracts that obtained the highest TI (after the statistical analysis) in comparison with the control is presented.

The differences among the obtained results can be related to the method that was used for the extraction of the antioxidant/bioactive substances, the size of the particles, the number of cycles during the extraction, the heating of extraction, and the solvent used in the extraction.^21^

In relation to the extraction method in the Soxhlet extractor that was used for extraction of the pecan nutshell, this one consisted of a traditional method, widely used for extractions. To grind the nutshells with a mortar and a pistil was used to make the particle size medium and thus to have a larger contact surface. However, using this maceration technique, it is not possible to have a uniform peel size, which can affect the interaction between the same and the solvent.

Regarding the time taken for extractions of the pecan nutshell, this was 4 h and therefore is within the limits of extraction time already studied by other authors, since this time should be in the range of 1 min up to 24 h.^21^ It should be noted that a very excessive period of extraction can lead to the oxidation of the phenolic compounds present before they pass into the solvent, in addition to the thermal decomposition of the phenolic compounds as a function of the heating during extraction, due to the interference in the stability of these compounds.^21^

With regard to the solvent used in the pecan shell extracts, it was possible to observe that there is a difference between the extracts used in the biodiesel washing. Among the three solvents used for the extraction of pecan nutshell, ethanol extract provided higher IT for biodiesel after washing compared to water and methanol + water (50/50). In the initial and optimized concentrations, the IT of the biodiesel washed with the ethanolic extract of the pecan nutshell 5 g L^−1^, was 9.93 and 9.45 h, respectively. It is worth mentioning that the IT required by the ANP for oxidative stability of biodiesel, at a temperature of 110 °C, is at least 8 h. Thus, soybean biodiesel washed with ENE5 presented oxidative stability results within the compliance required by the ANP.

However, during the process of washing the biodiesel with the ethanolic extract, the formation of a homogeneous mixture between the two was observed, and it was not possible to separate the extract from the biodiesel. Assuming that perhaps the antioxidant action reflected in the results of IT had occurred due to the presence of ethanol in biodiesel, the oxidative stability test was performed with a sample of biodiesel washed only with 95% ethanol without the pecan nutshell extract. The IT results obtained after this test were 0.91 and 1.14 h. In view of the above, it is suggested that ethanol is not responsible for the increase in the oxidative stability of the biodiesel, but rather the ethanolic extract of the pecan nutshell, showing the antioxidant action.

The aqueous extracts of 12 g L^−1^ pecan nutshell and the 12 g L^−1^ methanol and water extracts used in the biodiesel wash were at the concentrations that provided the highest IT for biodiesel, 7.40 and 7.37 h, respectively. Even though IT presented less than 8 h, the effect of adding them to the biodiesel washing process signals an increase in oxidative stability, which is verified by comparing these extracts with the control.

The solvents used for the extraction of the antioxidant substances of the pecan nutshell present different polarities, although the water, the ethanol, and the methanol are polar substances, there are significant differences of polarities in these substances, being that ethanol has the less polar characteristics.

The polarity of the solvents used in the extraction and/or their interaction with the phenolic compounds, phenolic acids, anthocyanins, tannins (substances that may be present in the pecan shell) and other nutrients,^16^ interfere directly in the extraction of the bioactive compounds. The use of solvents of different polarities has already been used in other studies in the literature because preliminarily, it is not possible to select a method that provides greater efficiency to extract the bioactive compounds.^21^

In view of this, it can be observed that the results obtained in this work were opposed to the results expected by polar paradox theory, which reports that apolytic lipophilic antioxidants found effective results in polar medium and those hydrophilic antioxidants found as effective in apolar medium^19^, ^22^, ^23^ that is, it was expected that as the antioxidant was used without biodiesel (apolar) extracts of water and methanol (polar) would have higher efficiency and consequently higher TI. However, we observed that the antioxidant in ethanolic extract, which is less polar in relation to these solvents, showed higher efficiency when used in biodiesel (apolar), that is, the partition coefficient of the bioactive compounds extracted in less polar solvents (ethanol) solubility in biodiesel.

Thus, studies indicate that the oxidative activity is not always linked to the polarity of the solvents used for extraction, since the structural characteristics and the composition of the antioxidants can provide different effects.^19^ As there are no studies that identify and quantify phenolic compounds present in pecan nuts, the precise elucidation of responses to the effects of these antioxidants is complex. Studies show that, by means of a color analysis of the nutshell, samples with a tendency to red tones present a higher content of condensed tannins.^19^ Thus, it can be said that the extracts obtained in this work indicated the presence of condensed tannins, since they had a red coloration.

Moreover, the concentrations of the extracts of the antioxidant substances can vary the oxidative stability according to the medium in which they are used,^19^, ^22^, ^23^ as for example: comparing the concentrations for the same solvent, if some caveats are necessary. For biodiesel washed with ethanolic extracts pecan nutshell with a concentration greater than 5 g L^−1^, they had lower IT values when compared to the above‐mentioned concentration. This fact suggests that even using a larger amount of pecan nutshell mass per liter of the solvent, there is a maximum concentration that can be extracted of antioxidants by ethanol, which in this case is 5 g L^−1^. It is emphasized that in concentrations higher than 5 g L^−1^ the extraction of the antioxidant compounds occurs relatively faster, this causes the extract to be exposed to the extraction temperature for a longer time, which can lead to the degradation of the bioactive compounds, thus minimizing the antioxidant activity.

In the same way, for the biodiesel washed with aqueous pecan nutshell extract, the one that obtained the highest IT among the tested concentrations of 5 to 15 g L^−1^ was the biodiesel washed with the aqueous pecan nutshell extract of 12 g L^−1^, a value between the two concentrations. The same case is for biodiesel washed with the methanol + water pecan nutshell extract at the concentration of 12 g L^−1^, that is, it is suggested that there is a limiting concentration with respect to the efficiency of the antioxidant, most probably due to an effect pro‐oxidant from a given concentration, catalyzing the oxidation.

### 2.2 Physical–Chemical Characterization

After obtaining the ideal concentration of the extracts in the biodiesel wash, these were submitted to the physicochemical characterization in order to verify if the addition of antioxidant would interfere in the parameters of the biodiesel quality control. The results obtained are contained in **Table** **3**.

**Table 3:** Physicochemical characterization

Tests1^a)^	Limit	CONTROL4	ANE12	ENE5	MNE12
Flash point [°C], [min]	100	30	36	21	33
Specific mass [Kg m^−3^]	850–900	875	880	860	875
Visual color	^a)^	Yellowish	Yellowish	Yellowish	Yellowish
Appearance	C.F.I.^b)^	C.F.I.	C.F.I.	C.F.I.	C.F.I.
Hydrogenation potential	^a)^	9.2	10.2	9.2	9.75
Conductivity	^a)^	75.6	^c)^	101.3	71.3

^a)^ Values not established by the ANP for biodiesel

^b)^ The equipment did not detect values, and the detection range of the equipment is 0–2000 pS m^−1^, with a resolution of 1 pS m^−1^

^c)^ C.F.I—Clean and free of impurities.

Based on the values indicated in Table 3, we observed that the soybean biodiesel washed with the extracts and the control had a low flash point that did not comply with the standard of at least 100 °C. This may have occurred because agitation was not sufficient during the washing process to carry the methanol remnants used in the transesterification reaction. The flash point may decrease with the addition of minimal amounts of alcohol.^24^ Thus, the ethanolic extract that presented the highest ethanol addition, when it formed a homogeneous mixture with the biodiesel, had the lowest flash point.

The specific mass of biodiesel samples as observed in Table 6, are within the values stipulated by the ANP. The color and appearance tests performed showed yellowish coloration and a clear and free of impurities (C.F.I). Thus, these samples fit the ANP specifications.

As observed, the pH values tended to the basic medium, which indicates that the basic catalysis used in biodiesel production left traces that were not carried during the washing process of the same. This could have been avoided by using an acidic wash for the purpose of neutralizing biodiesel.

As for the results of the conductivity, it can be seen that washing was more efficient for the samples washed with ENA12 since it presented null values of conductivity. The biodiesel washing process tends to decrease the conductivity of the sample. It should be pointed out that the nonseparation between the ethanolic pecan nutshell extract and biodiesel, the impurities removal function and the catalyst residues was inefficient, contributing to the increase of the conductivity value.

### 2.3 Kinetic Calculations: Order of Oxidation Reaction

In order to understand whether or not there was an influence of the addition of pecan nutshell extract to the oxidation kinetics of biodiesel, the order of reaction of the same and of the control was first identified and, subsequently, the activation energy was determined for them. Based on these data it is possible to predict how long soybean biodiesel can be stored at a given set temperature.

The order of reaction was identified by constructing graphs of the conductivity as a function of time to the IT of each sample, at a temperature of 110 °C, after which a linear regression was performed, obtaining an equation of the line *y* = *a* + *bx*. The value of *R* indicates the linearity of the points, the closer to 1, the greater the linearity, which in this case will indicate the order of the reaction. The values of *R*, the linear coefficient and the angular coefficient of the graph of the generated graphs for the reactions are shown in **Table** **4**: zero order (Equation (4)), first order (Equation (5)), and second order (Equation (6)).

**Table 4:** Reaction order for the biodiesel samples at a temperature of 110 °C

Biodiesel Samples	Order of reation	Value of R	Intercept	Slope
CONTROL4	0	0.22568	1.04038	−8.17705 × 10^−5^
	1ª^)^	0.87555	0.2001	−1.98263 × 10^−4^
	2ª^)^	0.66689	−2.49646	0.00107
ANE12	0	0.82598	0.15073	−5.88374 × 10^−5^
	1ª^)^	0.97002	−1.78364	−7.19743 × 10^−5^
	2ª^)^	0.92562	3.15889	0.00105
ENE5	0	0.31435	6.68973	−2.76739 × 10^−4^
	1ª^)^	0.90166	1.9037	−2.0001 × 10^−4^
	2ª^)^	0.89848	−9.95475	0.00144
MNE12	0	0.26617	4.23229	−2.26849 × 10^−4^
	1ª^)^	0.89492	1.13092	−2.34649 × 10^−4^
	2ª^)^	0.86791	−13.87057	0.0026

It can be observed in Table 4, for the sample of biodiesel control the order reaction that presented *R*‐value closer to 1, was the first order with *R* = 0.87555. In other studies, the first‐order identification is also mentioned for B100 soybean biodiesel without the addition of antioxidants.

For the sample washed with the ANE12, the order of the reaction was of the first order, with value of *R* = 0.97002. For the washed sample with ENE5, the reaction was also of first order, with value of *R* = 0.90166. Finally, for the sample washed with MNE12 was also first order with *R* = 0.89492.

The results obtained in relation to the order indicate that the mechanism of the oxidation reaction is the same regardless of whether or not the antioxidant is used, that is, the addition of this antioxidant does not affect the mechanism but rather slows the oxidation reaction.

After the established order for all biodiesel samples, first‐order graphs were constructed for temperatures 90, 100, and 120 °C, since for the temperature of 110 °C these graphs have already been constructed, see **Table** **5**.

**Table 5:** Reaction order for first order of reaction for biodiesel samples at temperatures of 90, 100, 110, and 120 °C

Biodiesel samples	Order of reaction	Value of *R*	Intercept	Slope
CONTROL4	90	0.77507	−0.11444	−5.01741 × 10^−5^
	100	0.6405	−2.66396	−2.99495 × 10^−5^
	110	0.87555	0.2001	−1.98263 × 10^−4^
	120	0.89775	1.66052	−4.9512 × 10^−4^
ANE12	90	0.90347	−2.73343	−1.32435 × 10^−5^
	100	0.84946	0.44636	−8.30699 × 10^−5^
	110	0.97002	−1.78364	−7.19743 × 10^−5^
	120	0.94516	1.15527	−4.67665 × 10^−4^
ENE5	90	0.7311	−1.3834	−2.27856 × 10^−5^
	100	0.67953	−2.38363	−5.47096 × 10^−5^
	110	0.90166	1.9037	−2.0001 × 10^−4^
	120	0.88749	−0.02556	−2.91304 × 10^−4^
MNE12	90	0.62039	−1.36898	−2.10189 × 10^−5^
	100	0.76601	−1.66127	−7.68283 × 10^−5^
	110	0.89492	1.13092	−2.34649 × 10^−4^
	120	0.91178	0.99585	−7.07837 × 10^−4^

According to Equation (5), the value of the velocity constant (*k*) is equal to the negative of the coefficient of the first‐order equation, which is found in Table 5. As *k* depends on the temperature (*T*), it is expected that is higher as the temperature increases. This fact was observed for all samples tested. With the value of k and the Arrhenius equation (Equation (7)) it is possible to calculate the activation energy (*E*_a_).

### 2.4 Activation Energy and Stocking Time

**Figure 3:**
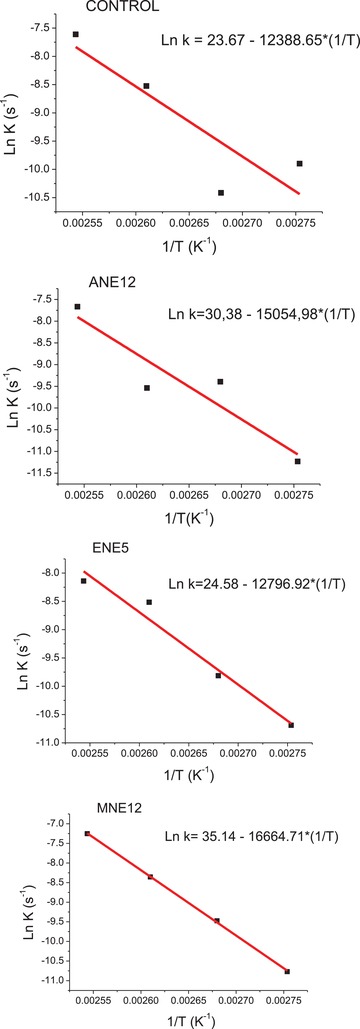
The graphs of activation energy of the Arrhenius equation for the control samples, ANE12, ENE5, and MNE12.

With the aid of the Arrhenius equation (Equation (7)), graphs were constructed with the values of the velocity constants calculated for the temperatures of 90, 100, 110, and 120 °C (Ln k versus 1/*T*) to elucidate the value of the activation energy. These graphs (**Figure** **3**) are shown in support. Since the value of the coefficient is equal to ‐*E*_a_/*R*, multiplying the value of the coefficient by ‐R gives the activation energy (Table 5).

As expected the activation energy for the control was lower than the other values found for the biodiesel samples that were washed with the pecan nutshell extract. This indicates that the addition of the antioxidant provided an increase in the *E*_a_, i.e., a higher energy for the oxidation process to occur, and that result is in line with what has already been previously exposed.

According to the studies of Borsato et al., the activation energy for soybean biodiesel (control) was close to 36 kJ mol^−1^ and with antioxidant (TBHQ) was close to 65 kJ mol^−1^, values substantially lower than those found for pecan nutshell extracts and control (**Table** **6**), such difference can be explained by the data treatment method.^8^ It should be noted that this study is relatively new and that the *E*_a_ data calculated using the conductivity data from the accelerated oxidation assay are sparse for comparison.

**Table 6:** Activation energy at temperatures of 90, 100, 110, and 120 °C

Biodiesel samples	Activation energy [Kg mol^−1^]	Stocking time [d]
CONTROL4	103.00	43
ANE12	125.17	263
ENE5	106.40	113
MNE12	138.55	50

**Figure 4:**
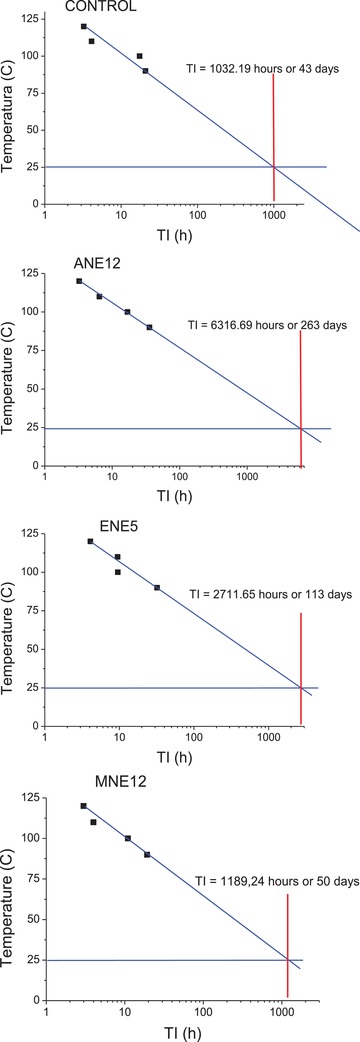
The graphs of stocking time the Arrhenius equation for the control samples, 1ANE12, ENE5, and MNE12.

In order to verify the period in which the biodiesel may be stocked or stored until oxidation reactions occur, it is of interest to determine the storage time thereof at room temperature, for example at 25 °C. For this, temperature graphs can be constructed as a function of IT, extrapolating the IT, as shown in graphs in **Figure** **4**.

The storage times presented in Table 6 how that, as expected, the samples with the addition of antioxidant obtained the highest storage times for biodiesel in comparison to the control sample. The biodiesel washed with the aqueous extract of pecan nutshell stands out among the others, as it obtained a storage time of 263 d.

## 3 Conclusions

The pecan nutshell extracts can be used as an antioxidant for soybean biodiesel. The concentrations of the extracts that provided the highest IT for the biodiesel after the washing process were the ethanolic extract of the pecan nutshell 5 g L^−1^ (9.45 h), the aqueous extract of the pecan nutshell 12 g L^−1^ (7.40 h) and the methanol + water extract of pecan nutshell 12 g L^−1^ (7.37 h).

The oxidation kinetics of the biodiesel washed in a conventional manner (control) and washed with the extracts of the pecan nutshell were first order, for all the samples tested.

The activation energy obtained for the control, for the biodiesel washed with the aqueous pecan nutshell extract 12 g L^−1^, for the biodiesel washed with the ethanolic pecan nutshell extract 5 g L^−1^ and for the biodiesel washed with the extract methanol + water of pecan nutshell 12 g L^−1^ were 103.00, 125.17, 106.40, and 138.55 kJ mol^−1^, respectively.

The extrapolated storage time for the control temperature of 25 °C for the biodiesel washed with the aqueous pecan nutshell extract 12 g L^−1^, for the biodiesel washed with the ethanolic pecan nutshell extract 5 g L^−1^ and for the biodiesel washed with the methanol + water extract of pecan nutshell 12 g L^−1^ were 43, 263, 113 and 50 d, respectively.

## 4 Experimental Section

*Reagents and Solutions*: The reagents and solutions used in this study were:

‐ Soybean oil commercial;
‐ Methyl alcohol 99.8% (CH_4_O)—methanol;
‐ Potassium hydroxide 85% (KOH);
‐ Deionized water;
‐ Ethyl alcohol 95% (C_2_H_6_OH)—ethanol;
‐ Alcoholic potash solution 10% (m/v).

*Soybean Biodiesel Production*: For the production of biodiesel, the transesterification reaction of the soybean oil with methanol was carried out by the alkaline route (KOH). First the oil was heated to 80 °C and simultaneously in another vessel the catalyst (KOH) was dissolved in methanol, warming this solution to 40 °C in the proportions of 1:0.01:0.3 (v/g/v) respectively. When the stipulated temperature was reached, the mixture called methoxy (methanol + KOH) was transferred to the vessel containing the soybean oil at 80 °C. Subsequently, the temperature of the mixture was maintained at 60 °C for 1 h under stirring to ensure high efficiency in the transesterification process.^25^ After the biodiesel production process, it was decanted for a period of 24 h, so that the glycerol was separated from the biodiesel. Next, the biodiesel was washed so that the reaction residues were removed by extraction.

*Cleaning of Biodiesel*: After biodiesel is produced and separated from glycerol, it is not free of catalyst and glycerol residues that influence some of its physicochemical properties. In this way the biodiesel must be washed, so that by extraction processes the residues are transferred from the organic phase to the aqueous phase, as shown in Figure 2. Normally the biodiesel washing process is done with 30% of the total volume of biodiesel, in a conventional way, in which the water is added to the biodiesel, under slight agitation. This biodiesel + water mixture is conditioned in a funnel of decanting for 24 h, for total separation of the aqueous phase and the biodiesel. Because water is a polar substance, it has the ability to extract/remove residues from alcohol, hydroxide used and glycerol remnants that remained in biodiesel after production.

For this study, the natural antioxidant study obtained from the extracts of the pecan nutshell was carried out by means of the biodiesel washing process. Thus, in addition to this conventional way of washing the biodiesel, different types of washing were used, which will be described later.

*Obtaining the Extracts of Pecan Nutshell*: Pecan nutshells were obtained from Pecantea Divinut Company, Cachoeira do Sul, Rio Grande do Sul, Brazil. To obtain extracts of the pecan nutshell, they were first washed and dried in a desiccator under vacuum at 600 atm and then milled with the aid of a mortar and a pistil. The extracts were obtained using a Soxhlet extractor using different solvents, for 4 h. The initial concentrations of the extracts were 5, 10, and 15 g L^−1^, with their respective solvents. Based on the results of obtained and with the statistical analysis of the data, an experimental design was carried out in which extracts with different concentrations were obtained for each solvent used in the nutshell extraction process, in order to optimize the results of the initial concentrations.

*Obtaining Biodiesel + Antioxidant Mixtures*: In order to obtain the biodiesel blends with the natural antioxidant, the shell extracts were added to the biodiesel (B100) in the washing process. Thus, in addition to the conventional washing, the process of washing the biodiesel with the shell extracts was also carried out. Therefore, the extracts served as antioxidant for biodiesel and also contributed to remove the production residues present in it.

In this work the samples were divided in two ways, according to the biodiesel washing process: the conventional and the presence of the extracts of the pecan nutshell. These biodiesel samples were identified as control and were parameters of comparison for the results.

In the second type of biodiesel washing, the same was carried out in the presence of the extracts of the pecan nutshell and followed the same steps reported for conventional washing, however the only difference was that instead of using water for washing, the solutions of the pecan nutshell extracts were used in their different concentrations and solvents.

*Oxidation Stability*: This assay was carried out with the purpose of verifying the antioxidant efficiency in the oxidation reaction of biodiesel. In order to measure the oxidation of biodiesel, the accelerated oxidation method, also known as the Rancimat test, was used in accordance with ANP regulations following the EN 14112 standards of resolution 45, DE 25.8.2014.^11^

The test was performed on the Rancimat 873 equipment, at a constant temperature of 110 °C, using samples of 3 g of soybean biodiesel, under accelerated heating, with an air inflation rate of 10 L h^−1^, to determine the induction period using 873 Biodiesel Rancimat Software 1.1, by the second derivative of the curve.^26^ The cleaning of the test tubes in which the samples were placed for the accelerated oxidation test was carried out with a 10% (m/v) alcoholic potash solution. This solution was prepared with 1.0 L of 95% ethanol, 120 g of potassium hydroxide and 120 mL of distilled water.^27^

*Physical–Chemical Characterization of Biodiesel*: The samples were submitted to analyze of flash point, specific mass, color, appearance, hydrogenation potential (pH), electrical conductivity, and ester content, and these tests were performed in triplicate.

*Flash Point*: The flash point analyzes were performed using a PENSKY‐MARTENS flash point equipment, coupled with a thermometer with a scale of 0 to 200 °C. The standard to be used was ASTM D92.^11^

*Especific Mass*: The specific mass tests at 20 °C were performed using an Incoterm brand densimeter, graduated rod ranging from 0.800 to 0.900 g cm^−3^. The method consisted in measuring the temperature of the system and the value established by the densimeter and after that the temperature was corrected to obtain the specific mass at 20 °C. This method is named ASTM D1298 and NBR 7148.^11^

*Visual Color and Appearance*: The determinations of the visual color and appearance of the samples were made in a 1L beaker, with the sample against light carefully observing: the presence of impurities, and/or water in the bottom of the container and the coloring of the product,^11^ and last one is expressed the visual color. For the appearance tests it can be classified as:

‐ Clean and free of impurities (C.F.I)
‐ Clear and with impurities (C.W.I)
‐ Turbid and free from impurities (T.F.I)
‐ Turbid and with impurities (T.W.I)

*Hydrogen Ionic Potential (pH)*: The pH measurements were performed by means of an assay using a digital Ph meter with bench top meter and calibration check, of the HANNA brand model HI 2221.^11^

*Electrical Conductivity*: The electrical conductivity was obtained using a Digimed device, model DM‐3P‐PE2, following the standard ASTM D2624, as foreseen in the resolution ANP N° 50, of 23 December 2013.^28^

*Experimental Planning and Statistical Analysis*: During the development of this work, three experiments were carried out using a completely randomized experimental design (DIC) with two replicates. The first two served as a basis for the definition of solutions to be used in the third and can be described as:

a) experiment consisting of seven treatments: i) control 1 (biodiesel washed in a conventional manner), ii) biodiesel washed with aqueous pecan nutshell extract (5 g L^−1^), iii) biodiesel washed with aqueous pecan nutshell extract (10 g L^−1^), biodiesel washed with pecan nutshell extract (15 g L^−1^), v) biodiesel washed with ethanolic pecan nutshell extract (5 g L^−1^), vi) biodiesel washed with ethanolic pecan nutshell extract (10 g L^−1^) vii) biodiesel washed with extract ethanolic pecan nutshell (15 g L^−1^) and,
b) Experiment composed of four treatments: i) control 2 (biodiesel washed in the conventional manner), ii) biodiesel washed with pecan nutshell extract + methanol + water (5 g L^−1^), iii) biodiesel washed with pecan nutshell extract methanol + water (10 g L^−1^) and iv) biodiesel washed with pecan nutshell extract methanol + water (15 g L^−1^).

From the results obtained from the IT (oxidative stability) in the two experiments above, and in order to verify the efficiency of pecan nutshell extracts that presented the best results as antioxidants for B100 soybean biodiesel, variance, and the Scott‐Knott's test for means comparison at 5% probability. The tests were done using the R software^29^ and the ExpDes package.^30^

Experiment (c) was defined from the results of experiments (a) and (b) and was composed of ten treatments: i) control 3 (biodiesel washed in the conventional manner), ii) biodiesel washed with aqueous pecan nutshell extract (8 g L^−1^), iii) biodiesel washed with aqueous pecan nutshell extract (10 g L^−1^), iv) biodiesel washed with aqueous pecan nutshell extract (12 g L^−1^), v) biodiesel washed with ethanolic pecan nutshell extract (3 g L^−1^), vi) biodiesel washed with pecan nutshell extract ethanolic (5 g L^−1^), vii) biodiesel washed with ethanolic pecan nutshell extract (7 g L^−1^), viii) biodiesel washed with pecan nutshell extract methanol + water (8 g L^−1^), ix) biodiesel washed with pecan nutshell extract methanol + water (10 g L^−1^) and x) biodiesel washed with pecan nutshell extract methanol + water (12 g L^−1^).

Again, in order to verify the efficiency of pecan nutshell extracts that presented the best results as antioxidants for B100 soybean biodiesel, analysis of variance and the Scott‐Knott's test were performed to compare means at 5% probability. These tests were performed using the R software^29^ and the ExpDes package^30^ and the results served as the basis for the final conclusion of the work.

*Study of Oxidation Kinetics*: The study of oxidation kinetics consisted in determining kinetic parameters, such as the order of the reaction and the activation energy of the samples with and without the addition of antioxidants. To elucidate these parameters were used the data from the oxidative stability test at temperatures of 90, 100, 110, and 120 °C. Thus, this study provides information on how quickly reagents are consumed and what products are formed and understand the mechanism of the reaction.^31^ In order to express a given concentration over time, the integrated velocity laws,^31^ given in Equations (1), (2), and (3), can be used.

Equation (1): Zero order for integrated speed law

(1) $$\left[A\right]={\left[A\right]}_{o}-kt$$

Equation (2): First order for integrated speed law

(2) $$\ln\left[A\right]=\ln{\left[A\right]}_{o}-kt$$

Equation (3): Second order for integrated speed law

(3) $$\frac{1}{\left[A\right]}=\frac{1}{{\left[A\right]}_{o}}+kt$$

where [*A*] is the concentration of the reagent of interest, [*A*]_0_ is the initial concentration of the reagent of interest, *k* is the rate constant, and *t* is the time. As it is not always possible to directly measure the concentration of biodiesel, it is possible to use conductivity values obtained in the oxidative stability test performed in rancimat as an indirect measure of this concentration. The lower the conductivity during the oxidation reaction the higher the concentration of biodiesel. As biodiesel oxidizes the conductivity increases and the concentration of biodiesel decreases. Thus, in the oxidation reaction of the biodiesel the conductivity (Λ) is inversely proportional to the concentration1. Thus the laws of integrated velocities can be modified, according to Equations (4), (5) and (6).

(4) $$\frac{1}{\left[\Lambda \right]}=\frac{1}{{\left[\Lambda \right]}_{o}}-kt$$

Equation (4): Zero Order for Biodiesel Degradation Reaction

(5) $$\ln\frac{1}{\left[\Lambda \right]}=\ln\frac{1}{{\left[\Lambda \right]}_{o}}-kt$$

Equation (5): First order for biodiesel degradation reaction

(6) $$\left[\Lambda \right]={\left[\Lambda \right]}_{o}+kt$$

Equation (6): Second Order for Biodiesel Degradation Reaction

The definition of the order of reaction is of great importance in order to understand the mechanism of reaction. From the establishment of the order of reaction, the activation energy (*E*_a_) can be obtained for the biodiesel oxidation process using the Arrhenius equation, Equation (7), and thus to investigate the performance of the antioxidant in biodiesel.^31^

(7) $$\ln k=\ln A-\frac{Ea}{R}\frac{1}{T}$$

Equation (7): Arrhenius equation

## Conflict of Interest

Patent deposit: INPI‐National Institute of Industrial Property in Brazil, under the number BR 10 2016 024243 6.
